# Low-Volume High-Intensity Interval Training in a Gym Setting Improves Cardio-Metabolic and Psychological Health

**DOI:** 10.1371/journal.pone.0139056

**Published:** 2015-09-24

**Authors:** Sam O. Shepherd, Oliver J. Wilson, Alexandra S. Taylor, Cecilie Thøgersen-Ntoumani, Ahmed M. Adlan, Anton J. M. Wagenmakers, Christopher S. Shaw

**Affiliations:** 1 Research Institute for Sport & Exercise Sciences (RISES), Liverpool John Moores University, Liverpool, United Kingdom; 2 Institute for Sport, Physical Activity & Leisure, Carnegie Faculty, Leeds Beckett University, Leeds, United Kingdom; 3 UBSport Hi Performance Centre, University of Birmingham, Birmingham, United Kingdom; 4 School of Psychology & Speech Pathology, Curtin University, Perth, Australia; 5 Department of Cardiology, Heartlands Hospital, Birmingham, United Kingdom; 6 School of Exercise & Nutrition Sciences, Deakin University, Geelong, Victoria, Australia; West Virginia University School of Medicine, UNITED STATES

## Abstract

**Background:**

Within a controlled laboratory environment, high-intensity interval training (HIT) elicits similar cardiovascular and metabolic benefits as traditional moderate-intensity continuous training (MICT). It is currently unclear how HIT can be applied effectively in a real-world environment.

**Purpose:**

To investigate the hypothesis that 10 weeks of HIT, performed in an instructor-led, group-based gym setting, elicits improvements in aerobic capacity (VO_2max_), cardio-metabolic risk and psychological health which are comparable to MICT.

**Methods:**

Ninety physically inactive volunteers (42±11 y, 27.7±4.8 kg.m^-2^) were randomly assigned to HIT or MICT group exercise classes. HIT consisted of repeated sprints (15–60 seconds, >90% HR_max_) interspersed with periods of recovery cycling (≤25 min.session^-1^, 3 sessions.week^-1^). MICT participants performed continuous cycling (~70% HR_max_, 30–45 min.session^-1^, 5 sessions.week^-1^). VO_2max_, markers of cardio-metabolic risk, and psychological health were assessed pre and post-intervention.

**Results:**

Mean weekly training time was 55±10 (HIT) and 128±44 min (MICT) (*p*<0.05), with greater adherence to HIT (83±14% vs. 61±15% prescribed sessions attended, respectively; *p*<0.05). HIT improved VO_2max_, insulin sensitivity, reduced abdominal fat mass, and induced favourable changes in blood lipids (*p*<0.05). HIT also induced beneficial effects on health perceptions, positive and negative affect, and subjective vitality (*p*<0.05). No difference between HIT and MICT was seen for any of these variables.

**Conclusions:**

HIT performed in a real-world gym setting improves cardio-metabolic risk factors and psychological health in physically inactive adults. With a reduced time commitment and greater adherence than MICT, HIT offers a viable and effective exercise strategy to target the growing incidence of metabolic disease and psychological ill-being associated with physical inactivity.

## Introduction

Leading a physically active lifestyle is associated with a greater exercise capacity and a reduced risk of developing type 2 diabetes and cardiovascular disease [1, 2]. Regular physical activity also contributes to improved mental health and well-being, by preventing [3] or alleviating depressive symptoms [4], increasing vitality and positive affect [5], and reducing subjective feelings of fatigue [6]. Nevertheless, the majority of the worldwide population fail to reach the recommended ≥150 minutes per week of moderate intensity exercise [7] and a “lack of time” is the most highly cited barrier to participation in sufficient physical activity [8]. Therefore, it would be beneficial to develop exercise strategies that maximise exercise adaptations, require a lower time commitment than the current guidelines, and offer a feasible and attractive exercise mode for the general population.

High intensity interval training (HIT) is an emerging exercise approach that may help to combat the plethora of chronic diseases associated with physical inactivity. HIT typically involves repeated bouts of high intensity exercise interspersed with periods of low intensity recovery or rest. However, the term ‘HIT’ encompasses a number of different high intensity interval exercise protocols. Broadly, HIT can be subdivided into sprint interval training (SIT; ≤30 second sprints at ≥100% VO_2max_), modified low-volume HIT (≤60 second intervals at >90% maximum heart rate) and aerobic interval training (4 minute intervals at 80–100% maximum heart rate) [9]. A growing number of small, tightly controlled laboratory-based studies demonstrate that both SIT and low-volume HIT promotes significant improvements in aerobic exercise capacity, blood pressure, vascular function and whole-body insulin sensitivity in sedentary and obese individuals and type 2 diabetes patients (reviewed in [10, 11]). Importantly, the magnitude of improvements in aerobic exercise capacity and other key cardio-metabolic risk factors following sprint interval training appear to be similar to moderate-intensity continuous training (MICT) [12–14], despite only requiring a fraction of the total energy expenditure and a vastly reduced total time commitment. Therefore, sprint interval training and/or low-volume HIT may provide an effective and time-efficient alternative to traditional MICT-based interventions to improve metabolic health.

Positive psychological responses to exercise (such as favourable changes in positive affect) are critical for sustained exercise participation (i.e., adherence; [15]), which in turn is needed to achieve long-term health benefits. To date, only acute psychological responses to HIT in young healthy males have been reported, and with conflicting results [16, 17]; enjoyment is greater [16] or similar [17] when comparing HIT and continuous exercise. In addition, HIT is associated with greater levels of fatigue following exercise and less pleasant affect during the exercise bout [17]. Importantly, the psychological effects of HIT have not been considered in sedentary individuals or examined over a longer-term (chronic) HIT intervention. Furthermore, given that affective responses reflect only one dimension of well-being, consideration should also be given to cognitive self-construal satisfaction [18] and vitality [19]; however, previous research examining the effects of acute HIT have not measured these dimensions of well-being.

The current evidence regarding the efficacy of HIT in comparison to MICT originates from researcher-led laboratory-based investigations involving relatively small numbers of participants [11, 20]. Exercise intensity in these studies was tightly prescribed and either based on percentage of maximal heart rate or percentage of maximal aerobic workload and performed under close supervision [11, 20]. The application of low-volume HIT to ecologically valid or ‘real world’ settings has only been explored in one previous study [21], and therefore questions remain around the feasibility and effects of HIT interventions implemented on a larger scale beyond the confines of the laboratory [22]. With this in mind, the purpose of the present study was to translate the principles of HIT into a ‘real world’ setting to determine whether it could be feasibly adopted as an exercise strategy to improve measured health outcomes. To this end, the current intervention was led by a fitness instructor and performed in a group-based community gym setting.

We hypothesized that 10 weeks of HIT, performed in instructor-led, group-based exercise classes, would elicit significant improvements in aerobic capacity, markers of cardio-metabolic risk and psychological health in physically inactive individuals. Secondly, given the lower time commitment of the HIT intervention, we hypothesised that adherence to the HIT intervention would be superior compared to MICT.

## Method

### Participants

Ninety healthy, inactive individuals, aged 18–60 years were recruited into the study and were employees of the University of Birmingham and Queen Elizabeth University Hospital Birmingham (for characteristics see Table 1). The study was approved by the University of Birmingham Research Ethics Committee. Following a verbal and written explanation of the nature and risks involved in the study, written, informed consent was obtained from all volunteers. Prior to entering the study, all subjects underwent a resting 12 lead electrocardiogram that was interpreted by a medical professional to detect abnormalities suggestive of underlying cardiovascular disease. Accordingly, all subjects entering the study were healthy (free of any known metabolic or cardiovascular disease) and did not meet the current physical activity guidelines (assessed through the completion of the Paffenbarger Physical Activity Questionnaire [23]) in the preceding year.

**Table 1:** Subject characteristics, body composition, exercise capacity and cardiovascular-related outcomes before and after HIT or MICT.

	HIT		Mean diff.	MICT		Mean diff.	Time effect	Time x group
Variable	*Pre*	*Post*	(95% CI)	*Pre*	*Post*	(95% CI)	*(p value)*	*(p value)*
*n* (males/females)	46 (15/31)	42 (12/30)		44 (15/29)	36 (14/22)			
Age (years)	42±11			43±11				
Body composition								
Height (m)	1.68±0.09			1.67 ± 0.01				
Body mass (kg)	78.8± 18.3	77.8±17.9	-1.0 (-1.6 to -0.3)	77.5±15.8	76.6±15.8	-0.9 (-1.6 to -0.2)	<0.001	0.88
BMI (kg.m^-2^)	27.7±5.0	27.4±4.9	-0.3 (-0.5 to -0.1)	27.7±4.6	27.4±4.7	-0.3 (-0.6 to -0.1)	0.001	0.94
Fat mass (kg)	26.0±10.2	25.2±9.9	-0.8 (-1.5 to -0.1)	24.7±8.5	23.7±9.5	-1.0 (-1.7 to -0.3)	<0.001	0.23
Muscle mass (kg)	50.8±13.0	50.6±12.5	-0.2 (-0.8 to 0.3)	49.7±11.5	49.9±11.3	0.2 (-0.3 to 0.8)	<0.001	0.06
Fat mass (%)	31.6±7.9	30.8±7.5	-0.8 (-1.5 to -0.1)	32.0±7.6	30.9±8.7	-1.0 (-1.7 to -0.3)	<0.001	0.40
Muscle mass (%)	65.0±7.6	65.6±7.3	0.6 (0 to 1.2)	64.7±7.4	65.8±8.4	1.1 (0.5 to 1.7)	0.71	0.07
Trunk fat (kg)	13.2±5.1	12.8±4.9	-0.4 (-0.9 to 0)	13.2±4.4	12.6±4.9	-0.6 (-1.1 to -0.1)	0.001	0.17
Leg fat (kg)	9.0±2.9	8.8±2.9	-0.2 (-0.4 to 0)	8.9±3.5	8.7±3.7	-0.2 (-0.4 to 0)	0.02	0.79
Exercise capacity								
VO_2 max_ (L.min^-1^)	2.50±0.77	2.71±0.78	0.21 (0.16 to 0.27)	2.43±0.70	2.59±0.75	0.16 (0.10 to 0.22)	<0.001	0.33
VO_2 max_ (mL.min^-1^.kg^-1^)	32.0±7.0	34.8±7.1	2.8 (2.0 to 3.6)	31.5±7.0	33.9±7.5	2.4 (1.7 to 3.3)	<0.001	0.90
W_max_ (W)	172±53	189±53	17 (13 to 22)	169±43	190±55	21 (16 to 26)	<0.001	0.34
Cardiovascular responses								
Systolic BP (mmHg)	123±11	123±10	0 (-1 to 3)	127±14	123±13*	-4 (-7 to -2)	0.02	0.01
Diastolic BP (mmHg)	76±10	75±9	-1 (-3 to 1)	78±9	76±9	-2 (-4 to 0)	0.03	0.64
MAP (mmHg)	92±9	91±9	-1 (-2 to 1)	95±10	92±17	-3 (-5 to -1)	0.03	0.09
AI_x_@75bpm	16±14	15±13	-1 (-2 to 1)	18±12	17±12	-1 (-2 to 1)	0.12	0.66
Resting HR (bpm)	67±9	64±9	-3 (-4 to -1)	67±9	65±10	-2 (-4 to -1)	0.01	0.96

Data provided are means ± SD. *BMI*, body mass index; *HR*, heart rate; *MAP*, mean arterial pressure; *W*_*max*_, maximum workload capacity.

*Indicates significant change from *pre* (*p*<0.05), when a training × group interaction was observed.

### Pre-training experimental procedures

**Fig 1:**
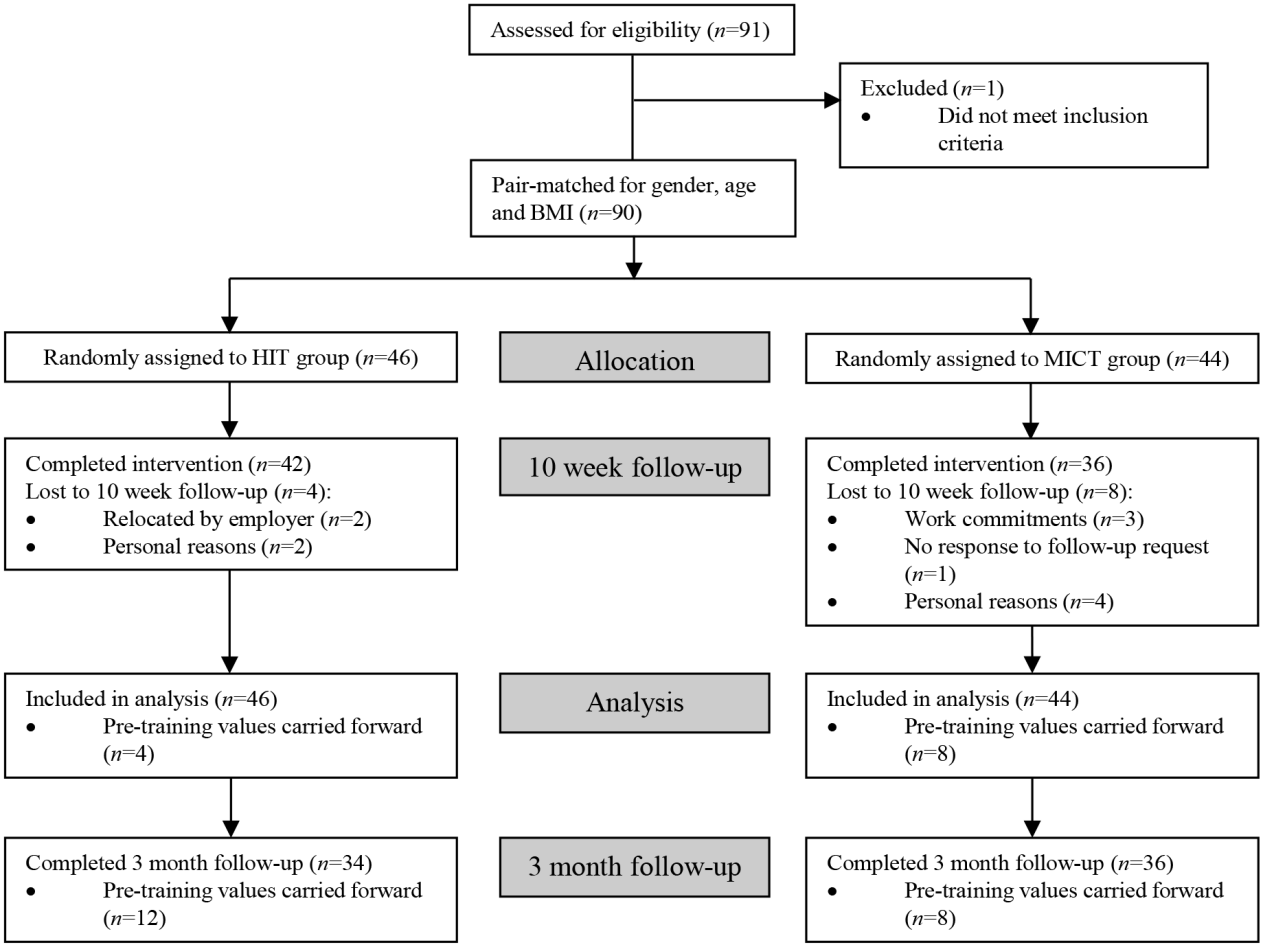
Flow chart of the study design. *BMI* body mass index, *HIT* high intensity interval training, *MICT* moderate-intensity continuous training.

Participants initially performed a progressive exercise test to volitional exhaustion on an electronically braked cycle ergometer (Lode BV, Groningen, The Netherlands) to determine maximal oxygen uptake (VO_2max_), as previously described [14]. On a separate day (>48 hours following the VO_2max_ test), subjects reported to the laboratory after an overnight fast (>10 hours), having completed a diet diary outlining their food intake in the preceding 24 hours period, which was collected and checked upon arrival. Following 15 minutes of supine rest, brachial artery blood pressure was measured, followed by an assessment of systemic arterial stiffness, as previously described [13]. A 2 hour oral glucose tolerance test (OGTT) was performed, as described [14], to assess insulin sensitivity. Body composition was assessed using a single frequency bioimpedance device (Tanita BC 418 MA Segmental Body Composition Analyzer, Tanita, Japan). On completion of the pre-training experimental procedures, subjects were stratified into sub-groups according to gender, age (under/over 40 years) and BMI (under/over 27 kg.m^-2^). Within each sub-group subjects were then placed into pairs with one member from each pair randomly assigned to either HIT or MICT (Fig 1).

### Training interventions

Exercise sessions took place in a dedicated cycle suite at the University of Birmingham sports centre in groups of 10–15 volunteers. Exercise training was carried out on mechanically-braked spinning bikes (Star Trac UK Ltd., Buckinghamshire, UK) commonly found in commercial gyms, that permitted the subject to manually adjust the fly-wheel resistance, thereby controlling the exercise intensity at which they were working. All exercise sessions were led by an instructor trained in delivering exercise classes. Each subject was provided with individual heart rate target values (determined from the results of the VO_2max_ test) to achieve during the exercise sessions. Both training groups (HIT or MICT) were asked to attend 3 supervised exercise sessions per week for 10 weeks and 5 sessions (Monday-Friday) were made available at equivalent times during the day in order to maximise attendance. A record of attendance was maintained throughout the 10 weeks by an investigator. All subjects were asked to maintain their habitual dietary and physical activity patterns during the training period.

#### High intensity interval training (HIT)

For each HIT training session subjects were provided with a heart rate monitor that transmitted heart rate to a central unit (Polar Team 2, Polar Electro Ltd., Warwick, UK). Individual heart rate values were projected on to a screen to allow participants to track their heart rate throughout each exercise session. The 10 week HIT training protocol is outlined in S1 Table. Briefly, each HIT training session began with a short (5 minutes) warm up of low intensity cycling, after which subjects performed repeated high intensity sprints of between 15 and 60 seconds in duration, interspersed with periods of active recovery (45 to 120 seconds in duration). For the high intensity sprints, subjects were instructed to adjust the fly wheel resistance and pedal at a cadence that they perceived to elicit an intense effort. The heart rate feedback provided was used to allow the participants to self-adjust their ‘effort’ in subsequent intervals in order to achieve a heart rate equivalent to >90% HR_max_. After each high intensity sprint, participants were advised to lower/remove the fly-wheel resistance during recovery periods. Each session concluded with a 5 minute cool down period. Each HIT session lasted a total of 18–25 minutes, depending on the duration and number of the high intensity sprints and recovery periods. Participants were informed of the duration of each high intensity sprint by the instructor throughout each session.

#### Moderate-intensity continuous exercise training (MICT)

All participants randomised to the MICT group were provided with a heart rate monitor (RS400, Polar Electro Ltd., Warwick, UK) for the 10 week training period which was used to record heart rate throughout each exercise training session. The 10 week MICT protocol is outlined in S1 Table. Briefly, each MICT session began with a short warm up of low intensity cycling, after which participants performed moderate-intensity continuous cycling for 30 minutes (week 1) progressing to 45 minutes (week 10). During the steady state period, participants were instructed to adjust the resistance of the bike to achieve a heart rate equivalent ~70% HR_max_ (which is equivalent to ~65% of pre-training VO_2max_). Each session concluded with a short cool down. In addition to attending 3 supervised exercise sessions per week, subjects were asked to perform 2 unsupervised moderate-intensity exercise sessions (consisting of brisk walking, jogging, cycling, or elliptical cross training), such that a total of 5 exercise sessions were performed each week. Heart rate monitors were also worn for unsupervised exercise sessions and training diaries were completed.

### Post-training experimental procedures

Post-training VO_2max_ was assessed >48 hours (and no longer than 5 days) following the last training session. At least 48 hours following the post-training VO_2max_ test, participants reported to the laboratory after an overnight fast (>10 hours), and underwent identical assessments to those outlined in ‘Pre-training experimental procedures’. Prior to this visit, participants were provided with the pre-training diet diaries and were asked to replicate their diet in the preceding 24 hours.

### Psychological assessments

As part of the pre and post-training experimental procedures health perceptions, positive and negative affect, life satisfaction and subjective vitality were assessed using validated questionnaires. One item from the 36-item short-form Medical Outcomes Study (MOS) health survey (SF-36; [24]) was used to examine general perceptions of health with higher scores indicating more positive perceptions of health. The 20-item Positive and Negative Affect Schedule (PANAS; [25]) was used to assess positive and negative affect. Greater scores on both the positive and negative affect sub-scales suggest that subjects experience high levels of positive and negative affect. Life satisfaction was assessed using the 5-item Satisfaction with Life Scale (SWLS; [26]) with high scores referring to high levels of life satisfaction. Subjects rated their feelings of energy available to themselves (subjective vitality) within the last month using the 7 item subjective vitality scale [27]. Once again, high scores infer that the individual experience high levels of energy. These scales were all administrated before and after the intervention, as well as three months following the exercise intervention. Finally, the 12 item Exercise-Induced Feeling Inventory [28], measuring revitalization, tranquillity, positive engagement, and physical exhaustion, was used to assess acute mood responses to single exercise training sessions. Greater scores on each of these sub-scales mean that subjects report high levels of each of these mood states. The latter scale was completed immediately following an exercise class during week 4 and week 8 of the intervention.

### Physical activity

Changes in habitual physical activity patterns were assessed using the International Physical Activity Questionnaire (IPAQ; [29]) from which subjects were asked to estimate the amount of time spent in walking, moderate and vigorous intensity physical activity over the preceding 7 day period. The outcome was assessed in METs (units of metabolic equivalence) per week. This questionnaire was administered immediately before and after the training period, as well as 3 months following the completion of the exercise intervention.

### Blood samples analysis

Blood samples collected into vacutainers containing either sodium fluoride, EDTA or silica were centrifuged at 1000g and 4°C for 10 minutes. Aliquots of plasma (and serum) were frozen and stored at -80°C until analysis. An ILab-600 semi-automatic spectrophotometric analyser was used to determine plasma glucose during the OGTT and fasting serum non-esterified fatty acid (NEFA), triglyceride (TG), total cholesterol (TC), LDL-cholesterol (LDL-C) and HDL-cholesterol (HDL-C) concentrations, in combination with the appropriate assay kit (all obtained from Instrumentation Laboratory Ltd UK, Warrington, UK, except for the NEFA assay, which was obtained from Randox, London, UK). Plasma insulin concentrations were determined using a commercially available direct insulin ELISA kit (Invitrogen, UK). Area under the curve (in response to the OGTT) for glucose, insulin and NEFA was calculated using the conventional trapezoid rule. Insulin sensitivity was assessed using the homeostatic model assessment (HOMA) and Matsuda [30] insulin sensitivity index.

### Statistical analysis

All data were analysed using statistical analysis software (Statistical Package for the Social Sciences for Windows, version 20.0, Chicago, IL, USA). Results are expressed as means ± SD. Significance was set at the 0.05 level of confidence. Differences in adherence were assessed using an independent samples t-test. Using an intention-to-treat approach (using baseline observation carried forward when follow-up data were missing), mixed-design ANOVA analyses were conducted to examine differences in the training response between HIT and MICT for all physiological, psychological and behavioural outcome variables, with the between-subject factor ‘condition’ and the within-subject factor ‘time’. When Mauchly’s test of sphericity was significant the Greenhouse-Geisser epsilon correction was employed. Significant main effects or interactions were assessed using Bonferroni adjustment *post hoc* analysis. The 95% confidence intervals (CI) for mean absolute pairwise differences between time points were calculated using the t-distribution. Physiological outcomes were assessed pre and post-training, whereas psychological outcomes were assessed at three time points (pre, post and 3 months post-training). This approach was taken primarily to investigate whether the increase in physical activity (as part of the intervention) was maintained after the 10 week intervention was complete. Finally, a one-way MANOVA analysis was conducted to examine differences between the training conditions in exercise-induced feelings (revitalization, tranquillity, positive engagement and physical exhaustion) in weeks 4 and 8. The one-way MANOVA approach was used because no changes between weeks 4 and 8 were expected.

## Results

### Verification of exercise intensity

During the HIT sessions the average maximum heart rate obtained at the end of each interval was equivalent to 91±6% HR_max_, with no significant difference seen between bouts lasting 15, 30, and 60 seconds. Mean heart rate during the MICT sessions was equivalent to 72±5% HR_max_, which was equivalent to the heart rate achieved at 64±9% VO_2max_.

### Adherence

All data related to adherence are presented in Table 2. Adherence to the training intervention was significantly greater in the HIT group (83±14% prescribed sessions attended; *n* = 42) compared to the MICT group (61±15% of prescribed sessions attended; *t*_67.74_ = 4.51; *p*<0.001; *n* = 36). The total number of sessions undertaken by the MICT group (33±12) was, however, significantly greater than the number of sessions undertaken by the HIT group (25±5) (*t*_55.02_ = 3.96; *p*<0.001). This equated to an average of 2.6±0.4 sessions (of 3 prescribed) undertaken per week by the HIT group and 3.4±1.0 sessions (of 5 prescribed) per week by the MICT group. Over the training period, the MICT group attended 21±7 supervised sessions (70±18% of all exercise sessions) and performed 11±8 sessions unsupervised (30±18% of all sessions). When comparing only the number of supervised sessions attended, the HIT group attended significantly more sessions compared to the MICT group (25±5 vs. 21±7, respectively; *t*_76.51_ = 2.57; *p* = 0.012). The average weekly training time completed was over 2-fold greater in the MICT group (128±44 minutes) compared to the HIT group (55±10 minutes; *t*_43.63_ = 10.90; *p*<0.001). Overall, 4 and 8 people in the HIT (3 male, 1 female, 34±9 years, 27.3±3.0 kg.m^-2^, 37.7±6.8 ml.min^-1^.kg^-1^) and MICT (1 male, 7 female, 42±12 years, 27.0±4.3 kg.m^-2^, 29.1±4.6 ml.min^-1^.kg^-1^) groups, respectively, were lost to follow-up at 10 weeks (Fig 1). We used the baseline observation carried forward method to undertake intention-to-treat analysis, whereby the baseline values of the drop-outs were carried forward and included in the overall analysis.

**Table 2:** Attendance to prescribed exercise sessions and time spent exercising.

			Group effect
Variable	HIT	MICT	*(p value)*
Adherence (% of prescribed sessions completed)	83±14	61±15	<0.001
Total number of sessions completed	25±5	33±12	<0.001
Number of sessions completed (per week)	2.6±0.4	3.4±1.0	0.02
Number of supervised sessions completed	25±5	21±7	0.01
Number of unsupervised sessions completed	N/A	11±8	-
Total time spent training (min) per week (range)	55±10 (36–65)	128±44 (50–225)	<0.001

Data provided are means ± SD.

### Exercise capacity and body composition

**Fig 2:**
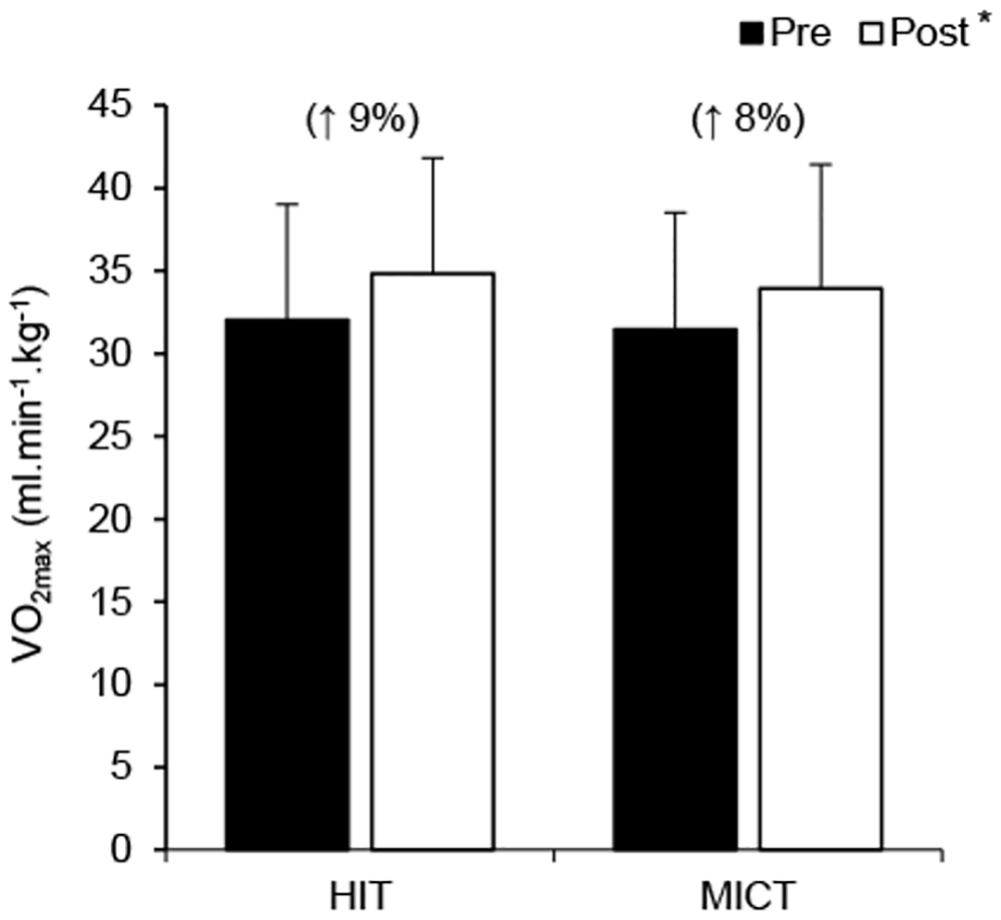
VO_2max_ response to HIT and MICT. Values are presented as means ± SD. Values in parentheses represent the mean percentage change from pre-training. *Main training effect (*p*<0.05).

All exercise capacity and body composition data are presented in Table 1 and Fig 2. No group differences were detected at baseline in any of the variables relating to exercise capacity or body composition (*p*>0.05). Results revealed a significant main effect of time for VO_2max_ (*F*(1, 88) = 86.72; *p*<0.001; *η*_*p*_ *²* = .50) and maximum workload capacity (*F*(1, 88) = 122.44; *p*<0.001; *η*_*p*_ *²* = .58), with increases post-training (VO_2max_: HIT 9±4%, MICT 8±3%), but no difference between groups. Training reduced body mass (*F*(1, 88) = 15.63; *p*<0.001; *η*_*p*_ *²* = .15) and BMI (*F*(1, 88) = 12.54; *p*<0.001; *η*_*p*_ *²* = .12), with no difference between groups (Fig 2). With regards to body composition, results revealed a main effect of time for a reduction in whole-body absolute fat mass (*F*(1, 83) = 17.11; *p*<0.001; *η*_*p*_ *²* = .17) and relative fat mass (*F*(1, 83) = 12.50; *p*<0.001; *η*_*p*_ *²* = .13), with no difference between groups. The reduction in fat mass occurred primarily in the trunk region, as a main effect of time was observed (*F*(1, 83) = 11.68; *p* = 0.001; *η*_*p*_ *²* = .12), with no difference between groups.

### Blood pressure and arterial stiffness

All blood pressure and arterial stiffness data are presented in Table 1. No group differences were detected at baseline in any of the variables relating to blood pressure or arterial stiffness (*p*>0.05). A significant group × time interaction was observed for systolic blood pressure (*F*(1, 87) = 10.55; *p* = 0.002; *η*_*p*_ *²* = .11), with a reduction in response to MICT only. Results revealed a trend for a main effect of time for diastolic blood pressure (*F*(1, 87) = 3.80; *p* = 0.05; *η*_*p*_ *²* = .04) and a significant main effect of time for mean arterial blood pressure (*F*(1, 87) = 4.85; *p* = 0.03; *η*_*p*_ *²* = .05), with a decrease following training but no difference between groups. A main effect of time was also detected for resting heart rate (*F*(1, 85) = 15.18; *p* = 0.01; *η*_*p*_ *²* = .15), with no difference between groups, whereas no significant changes in normalised augmentation index (AI_x_@75bpm) occurred following either HIT or MICT.

### Insulin sensitivity and blood lipids

**Fig 3:**
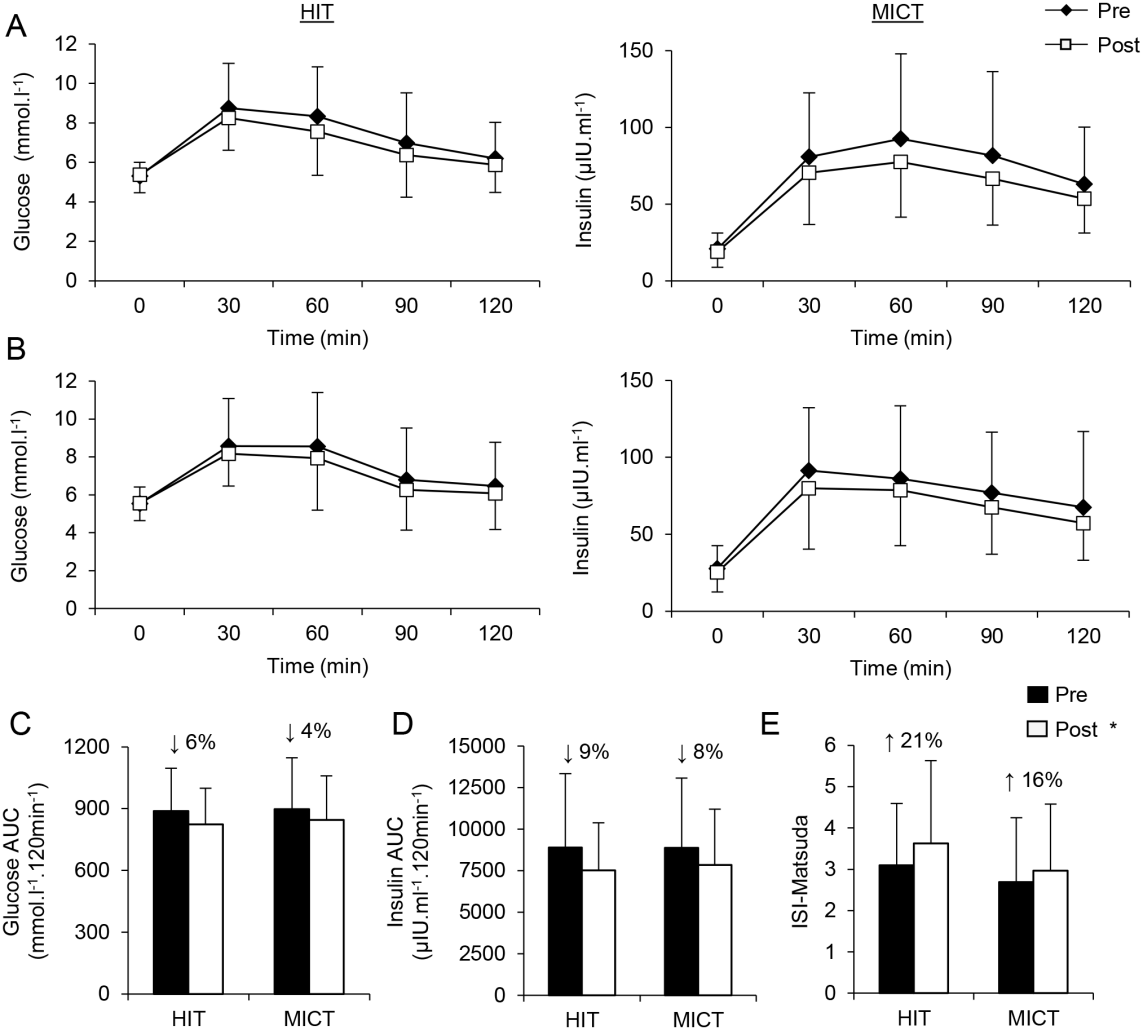
Oral glucose tolerance test responses. Plasma glucose *(A)* and insulin *(B)* concentrations during an oral glucose tolerance test, including corresponding total area under the curve (AUC, *C* and *D*, respectively) before and after HIT or MICT. Changes in insulin sensitivity as determined using the Matsuda insulin sensitivity index in response to HIT and MICT *(E)*. Values are presented as means ± SD. *Main training effect (*p*<0.05). Values in parentheses represent the mean percentage change from pre-training.

Insulin sensitivity and blood lipid concentrations are presented in Table 3 and Fig 3. No group differences were detected at baseline in any of the variables relating to insulin sensitivity or blood lipids (*p*>0.05). Training reduced fasting plasma insulin (*F*(1, 87) = 7.23; *p* = 0.01; *η*_*p*_ *²* = .08), whereas fasting plasma glucose was unchanged (*p* = 0.68), with no difference between groups. Notably, training reduced both plasma glucose area under the curve (AUC) (*F*(1, 86) = 17.36; *p*<0.001; *η*_*p*_ *²* = .17) and plasma insulin AUC (*F*(1, 86) = 25.71; *p*<0.001; *η*_*p*_ *²* = .23) during the OGTT, with no difference between groups (Fig 3C and 3D). An increase in insulin sensitivity was observed using both HOMA (*F*(1, 86) = 6.48; *p* = 0.013; *η*_*p*_ *²* = .07) and Matsuda (*F*(1, 86) = 14.18; *p*<0.001; *η*_*p*_ *²* = .14) indices of insulin sensitivity, with no difference between groups (Fig 3E). Training reduced fasting NEFA concentrations (*F*(1, 87) = 6.87; *p* = 0.01; *η*_*p*_ *²* = .07), whereas a trend for a reduction in fasting TG concentration was observed following training (*F*(1, 87) = 3.87; *p* = 0.052; *η*_*p*_ *²* = .04), with no difference between groups (Table 3). Total cholesterol (*F*(1, 87) = 14.54; *p*<0.001; *η*_*p*_ *²* = .14) and LDL-C (*F*(1, 87) = 10.72; *p* = 0.01; *η*_*p*_ *²* = .11) were also lower following training, whereas HDL-C concentrations were significantly increased (*F*(1, 87) = 5.61; *p* = 0.03; *η*_*p*_ *²* = .06), with no difference between groups (Table 3). Accordingly, the LDL-C to HDL-C ratio was reduced following training (*F*(1, 87) = 19.16; *p*<0.001; *η*_*p*_ *²* = .18), with no difference between groups (Table 3).

**Table 3:** Fasting glucose, insulin and lipids before and after HIT or MICT.

	HIT		Mean diff.	MICT		Mean diff.	Time effect	Time x group
Variable	*Pre*	*Post*	(95% CI)	*Pre*	*Post*	(95% CI)	*(p value)*	*(p value)*
Fasting glucose (mmol.L^-1^)	5.3±0.7	5.4±0.9	0.1 (-0.1 to 0.3)	5.5±0.9	5.6±0.9	0.1 (-0.2 to 0.2)	0.68	0.94
Fasting insulin (μIU.mL^-1^)	20.8±10.4	19.1±10.2	-1.7 (-3.9 to 0.4)	27.8±14.8	25.3±12.8	-2.5 (-4.8 to -0.2)	0.01	0.52
ISI-HOMA	5.0±2.8	4.6±2.5	-0.4 (-1.0 to 0.2)	7.3±4.9	6.6±4.1	-0.7 (-1.3 to -0.1)	0.01	0.61
NEFA (mmol.L^-1^)	0.67±0.29	0.60±0.27	-0.07 (-0.13 to -0.01)	0.46±0.17	0.41±0.16	-0.05 (-0.12 to 0.01)	0.01	0.71
NEFA AUC (mmol.L^-1^.min^-1^)	30±13	26±13	-4 (-8 to -2)	23±9	21±7	-2 (-5 to 1)	0.01	0.23
TG (mmol.L^-1^)	1.22±0.49	1.09±0.48	-0.13 (-0.25 to -0.01)	1.34±0.84	1.29±0.78	-0.05 (-0.17 to 0.08)	0.05	0.34
Cholesterol (mmol.L^-1^)	5.1±1.2	4.8±0.9	-0.3 (-0.7 to -0.1)	5.4±1.3	5.0±1.4	-0.4 (-0.7 to -0.1)	0.01	0.79
LDL-C (mmol.L^-1^)	3.0±1.1	2.7±1.2	-0.3 (-0.6 to -0.1)	2.9±1.0	2.6±0.9	-0.3 (-0.5 to 0)	0.01	0.73
HDL-C (mmol.L^-1^)	1.36±0.38	1.40±0.37	0.04 (-0.06 to 0.16)	1.24±0.46	1.38±0.40	0.14 (0.03 to 0.25)	0.03	0.25
LDL-C:HDL-C ratio	2.5±1.3	2.1±1.2	-0.4 (-0.7 to 0)	2.9±1.9	2.2±1.2	-0.7 (-1.1 to -0.4)	<0.001	0.17

Data provided are means ± SD. *AUC*, area under the curve; *HOMA*, homeostatic model assessment; *ISI*, insulin sensitivity index; *NEFA*, non-esterified fatty acids; *OGTT*, oral glucose tolerance test; *TG*, triglyceride.

### Psychological outcomes

All data relating to psychological outcomes are presented in Table 4. No group differences existed at baseline in any of the psychological variables assessed (*p*>0.05).

**Table 4:** Psychological health and self-reported physical activity before and after HIT or MICT and at the 3-month follow-up.

	HIT			MICT			Time effect	Time x group
Variable (score range)	*Pre*	*Post*	*Follow-up*	*Pre*	*Post*	*Follow-up*	*(p value)*	*(p value)*
Perceptions of health (1–5)	2.96 ± 1.13	3.44 ± 1.10	3.27 ± 1.35	3.18 ± 1.21	3.56 ± 1.17	3.33 ± 1.45	<0.001	0.659
Positive affect (PANAS; 1–5)	3.60 ± 0.84	3.81 ± 0.92	3.57 ± 0.96	3.42 ± 0.89	3.89 ± 0.98	3.47 ± 1.02	<0.001	0.209
Negative affect (PANAS; 1–5)	1.95 ± 0.83	1.82 ± 0.83	1.88 ± 0.92	1.97 ± 0.92	1.77 ± 0.83	1.93 ± 0.92	0.050	0.726
Life satisfaction (1–7)	4.88 ± 1.66	4.91 ± 1.66	4.73 ± 1.94	4.49 ± 1.84	4.83 ± 1.84	4.40 ± 2.03	0.105	0.475
Subjective vitality (1–7)	4.10 ± 1.38	4.65 ± 1.38	4.26 ± 1.66	4.15 ± 1.48	4.73 ± 1.48	4.29 ± 1.75	<0.001	0.970
Walking (IPAQ; MET min.wk^-1^)	757 ± 2120	640 ± 2406	1148 ± 2591	1050 ± 2250	1311 ± 2554	1565 ± 2747	0.004	0.359
Moderate physical activity (IPAQ; MET min.wk^-1^)	213 ± 889	287 ± 843	586 ± 1971	289 ± 953	369 ± 907	468 ± 2117	0.118	0.575
Vigorous physical activity (IPAQ; MET min.wk^-1^)	335 ± 907	613 ± 1091	668 ± 1595	379 ± 953	734 ± 1146	685 ± 1677	0.020	0.874

Data provided are means ± SD.

#### Training effects

Using intention-to-treat analysis, mixed-design ANOVA analyses were conducted to examine differences between HIT and MICT on all psychological and behavioural outcome variables. The results revealed a main effect of time for perceptions of health (*F*(1.71, 140.01) = 10.99; *p*<0.01; *η*_*p*_ *²* = .12) with increases from pre- to post-training. Likewise, there was a significant time effect for positive affect (*F*(1.60, 133.08) = 13.12; *p*<0.01; *η*_*p*_ *²* = .14) with increases in positive affect as a result of training. Negative affect decreased in both groups as a result of training (*F*(1.71, 142) = 3.22; *p* = 0.05; *η*_*p*_ *²* = .04). However, pairwise comparison analyses revealed that these increases were not sustained at the three month follow-up. There were no changes over time nor any group differences in life satisfaction (*F*(1.71, 142.16) = 2.38; *p*>0.05; *η*_*p*_ *²* = .03). With regard to subjective vitality, results revealed a significant effect for time (*F*(1.80, 149.43) = 12.30; *p*<0.01; *η*_*p*_ *²* = .13), but no significant interaction effect (*p*>0.05). There was an increase in subjective vitality as a result of training, which decreased to baseline levels at follow-up.

#### Acute exercise-induced effects

The effects of single exercise sessions on exercise-induced feelings (revitalisation, positive engagement, tranquillity and exhaustion) were assessed immediately after the training sessions in weeks 4 and 8 in both groups. No changes were predicted between weeks 4 and 8, so a one-way MANOVA analysis was conducted for each week, entering all four exercise feeling states into the analysis. The analyses revealed no significant differences between the groups on the outcomes in either week 4 (Pillai’s Trace = .083; *F*(4, 68) = 1.54; *p*>0.05; *η*_*p*_ *²* = .08) or week 8 (Pillai’s Trace = .077; *F*(4, 68) = 1.42; *p*>0.05; *η*_*p*_ *²* = .08).

### Self-reported physical activity

As expected self-reported levels of vigorous physical activity increased as a result of training (*F*(1.65, 135.03) = 4.37; *p*<0.05; *η*_*p*_ *²* = .05) and the effects were similar in both groups (i.e., no significant interaction effect; *p*>0.05). Pairwise comparison analyses showed that the participants sustained these levels of vigorous physical activity at the 3-month follow-up. In contrast, there were no changes over time in self-reported moderate intensity physical activity (*F*(1.21, 98.95) = 2.42; *p*>0.05; *η*_*p*_ *²* = .03), nor any signification time × interaction effect (*p*>0.05). Walking increased over time in both groups (*F*(1.69, 140.50) = 6.43; *p*<0.01; *η*_*p*_ *²* = .07), but this increase was evident from post-intervention to follow-up, not during the intervention.

## Discussion

To date, small laboratory-based investigations have indicated that HIT can effectively improve cardiovascular and metabolic health. In an effort to increase the applicability of HIT to a wider population, we developed an exercise programme that can be delivered to large numbers of people with a wide range of age and physical fitness in a gym setting. As such, the delivery mode has fundamental differences to previous lab-based interventions where exercise intensity is strictly controlled and performed under close and constant supervision. With guidance and encouragement from the instructor, participants self-selected an exercise intensity which they perceived to elicit an intense effort. Using this approach, participants were able to achieve heart rates of ≥90% HR_max_ at the end of the high intensity intervals. Further, we varied the duration of the intervals in the HIT sessions between 15 and 60 s, as intervals spanning this duration have previously been shown to induce improvements in VO_2max_, glucose tolerance and related muscle and cardiovascular adaptations [12–14, 31, 32]. Importantly, the exercise programme was sufficient to induce significant cardiovascular and metabolic adaptations and psychological benefits that were as large as those achieved in the MICT group over the course of the 10 week intervention.

Group-based HIT improved VO_2max_ to a similar extent as MICT, despite the weekly training time commitment being less than half of that required for the MICT intervention. The ~9% improvement in VO_2max_ observed in the present study is at the top end of the 4–13% increase in VO_2max_ reported in a recent meta-analysis of laboratory-based studies primarily using ‘all-out’ sprint interval protocols [33]. This finding has clinical importance given that VO_2max_ is the strongest prognostic marker of cardiovascular mortality [34] and improvements in VO_2max_ are associated with a reduction in all-cause mortality risk [35]. Previous epidemiological studies report an 8–17% reduction in all-cause mortality for each 1-MET (~3.5 ml.min^-1^.kg^-1^) increase in exercise capacity [34]. Given that the average increase in VO_2max_ in the present study was ~3 ml.min^-1^.kg^-1^, we can conclude that instructor-led, group-based HIT induces clinically meaningful benefits to those who fail to achieve sufficient levels of physical activity.

A similar improvement in whole-body insulin sensitivity was also observed following group-based HIT compared to MICT when estimated using both the Matsuda and HOMA indices of insulin sensitivity. These findings are in agreement with previous controlled laboratory studies investigating low-volume HIT and SIT in inactive lean [14, 32] and overweight individuals [36]. As skeletal muscle accounts for approximately two-thirds of glucose disposal in response to an oral glucose load, it is likely that adaptations within skeletal muscle account for much of the observed improvement in whole-body insulin sensitivity. In support, short-term laboratory-based studies report increased mitochondrial biogenesis [12, 31], greater expression of oxidative enzymes [12, 14, 31], angiogenesis [13], and enhanced glycogen and intramuscular lipid storage [12, 14], adaptations which likely underlie much of the improvement in insulin sensitivity seen in the present study.

HIT and MICT both led to reductions in plasma NEFA, TC and LDL-C and an increase in HDL-C. Eight weeks of HIT has previously been shown to reduce TC and LDL-C, but did not increase HDL-C, in lean individuals [37]. A strong trend for a reduction in plasma TG concentration was also observed following training (*p* = 0.052). Closer inspection of the data indicates that this is driven by the decrease in TG following HIT (Table 3). This is the first study, to the authors’ knowledge, to observe a reduction in TG following low-volume HIT. This is important, since high fasting plasma TG concentrations are associated with a high cardiovascular disease risk [38]. The lack of change in TG in response to MICT in the present study is not immediately clear, but could be the consequence of the greater variation observed in TG concentrations in the MICT group (see Table 3), or the use of intention-to-treat analysis in which 8 baseline values were carried forward. Nevertheless, the improvements in aerobic capacity, whole body insulin sensitivity, and reduced adiposity in addition to the improvements in blood lipids following 10 weeks of group-based HIT will result in meaningful reductions in cardiovascular and metabolic disease risk.

The present study also represents the first examination of both chronic and acute psychological responses to HIT. With regard to chronic exercise effects, the results revealed beneficial effects of HIT on health perceptions, positive and negative affect, and subjective vitality. These findings are generally consistent with results of previous intervention studies examining the effects of aerobic-based training on such outcomes [39, 40]. Life satisfaction did not change over time in response to either HIT or MICT. Previous studies have also failed to show consistent effects on life satisfaction as a result of other exercise interventions, although such studies have mainly been conducted with older adults [41]. High levels of life satisfaction reported by all subjects at baseline (*M* = 4.49–4.88 out of a maximum score of 7) may partly explain our results. However, as life satisfaction is an important dimension of subjective well-being along with the presence of positive affect and the absence of negative affect, it can be tentatively concluded that HIT has stronger effects on affective, compared to cognitive, dimensions of subjective well-being.

None of the enhanced psychological responses were sustained at the 3-month follow-up. This is surprising, as we did observe that walking increased from post-intervention to the 3-months follow-up, while the increases in vigorous intensity physical activity as a result of the intervention were sustained at the follow-up. We suggest that the use of intention-to-treat statistical analysis, which provides a conservative test of intervention effects, is partly responsible for this finding.

Some acute psychological outcomes of HIT have been examined previously [16, 17]. In the present study, distinct feelings induced by an acute HIT bout were also examined post-exercise in weeks 4 and 8. The results revealed no differences between the HIT and MICT condition on revitalization, tranquillity, positive engagement or physical exhaustion. These results suggest again that HIT and MICT have a comparable ability to induce mood changes. Using a measure of fatigue, Oliveira *et al*. [17] found that subjects in the HIT condition reported greater levels of fatigue than the continuous exercise group post-exercise. This is in contrast to the finding in the present study that physical exhaustion, a conceptually similar outcome, did not differ between the HIT and MICT condition. This discrepancy is particularly noteworthy given that the subjects in the study by Oliveira *et al*. were young healthy males and not inactive at baseline. It is likely that the different training characteristics between the two studies (e.g. greater interval duration, treadmill-based exercise, and prescribed exercise intensity) may partly explain this discrepancy. Moreover, the fact that the responses in the present study were recorded within the context of an intervention and responses were observed after 4 and 8 weeks of training, by which the participants had become more accustomed to HIT, may also influence the results.

In the context of a “lack of time” being the most commonly reported barrier to physical activity, the potential time saving benefits of HIT are well documented. In the present study, adherence was greater with group-based HIT compared to MICT (~83 vs. ~61%, of sessions completed, respectively) and the number of drop-outs in the HIT group was lower than MICT (8 vs. 4, respectively). It is important to note that in order to mimic the current exercise guidelines, the MICT group were prescribed 5 exercise sessions per week versus the 3 sessions per week prescribed to the HIT group. Nevertheless, the lower adherence rates and higher drop-out rates in the MICT group indicates that 30–45 minutes of structured exercise performed 5 times per week was difficult to achieve for many of our subjects. This is in line with reports that approximately 50% of adults drop out of exercise programmes within the first six months [42] and is reflected by the low number of individuals who achieve the current physical activity recommendations of ≥150 minutes per week of moderate intensity exercise [7]. On the other hand, 3 sessions per week of HIT appeared to be more feasible and achievable for the participants recruited in the current study. Thus, the findings provide support for the feasibility of instructor-led, group-based HIT as a strategy to improve engagement in effective and regular physical activity in those who are currently inactive.

There are several limitations of the study that should be considered when designing future investigations. First, we only recruited healthy participants free of metabolic or cardiovascular disease, and therefore we cannot yet generalise our results to diseased populations. However, laboratory-based HIT has proved effective in improving health markers in people with type 2 diabetes [43] and patients with coronary artery disease [44], highlighting the potential for the ‘real world’ HIT intervention used in this study to also be effective in diseased populations. Second, we describe the effects of a 10 week HIT programme and the longest HIT interventions described to date are only up to 12 weeks in duration [21, 45]Therefore, we do not know the longer term (>12 weeks) effects of HIT on physiological and psychological responses and/or adherence rates in sedentary populations. Finally, the number of sessions that the participants were required to undertake each week differed between HIT and MICT (3 and 5 sessions, respectively). Therefore, we do not know if the higher adherence rates with HIT would remain if an equal number of sessions per week were prescribed for HIT and MICT. However, it should be noted here that both groups had the opportunity to attend the same number (three) of supervised sessions per week, yet attendance in the HIT group was still higher than the MICT group in these supervised sessions.

In summary, this study demonstrates for the first time that HIT induces wide-ranging health benefits encompassing improvements in cardiovascular and metabolic disease risk factors in addition to psychological health, and self-reported physical activity levels. These findings are particularly important considering that the majority of the population fail to achieve the recommended minimum of 150 minutes of moderate intensity exercise per week [46], partly due to a (perceived) lack of time, and are therefore at an elevated risk of developing metabolic and cardiovascular disease. This study presents convincing evidence that the application of HIT through instructor-led, group-based exercise classes in a gym setting provides a feasible and effective approach to increase exercise engagement and adherence to exercise behaviours. As such, this delivery mode of low-volume HIT offers a novel approach to target the growing incidence of metabolic disease and psychological ill-being associated with physical inactivity.

## Supporting Information

S1 Table Overview of HIT and MICT training protocols. (PDF)
