# Supplementary material for: Low-Volume High-Intensity Interval Training in a Gym Setting Improves Cardio-Metabolic and Psychological Health
Source: PLoS One. 2015 Sep 24;10(9):e0139056. doi: 10.1371/journal.pone.0139056 (PMC4581708; doi:10.1371/journal.pone.0139056)
Supplement: S1 Table — (PDF) [file pone.0139056.s001.pdf]

| | HIT | | MICT | |
|---|---|---|---|---|
| *Week* | *Session* | *Session time (min:sec)* | *Session* | *Session time (min:sec)* |
| 1 | 30 s HIE, 2 min recovery; repeat 4x | 20:00 | 20 min MICT | 30:00 |
| 2 | 15 s HIE, 45 s recovery; repeat 8x | 18:00 | 20 min MICT | 30:00 |
| 3 | 30 s HIE, 90 s recovery; repeat 5x | 20:00 | 25 min MICT | 30:00 |
| 4 | 60 s HIE, 60 s recovery; repeat 5x | 20:00 | 25 min MICT | 30:00 |
| 5 | 30 s HIE, 60 s recovery; repeat 7x | 20:30 | 30 min MICT | 35:00 |
| 6 | 15 s HIE, 45 s recovery; repeat 12x | 22:00 | 30 min MICT | 35:00 |
| 7 | 30 s HIE, 60 s recovery; repeat 9x | 23:30 | 35 min MICT | 40:00 |
| 8 | 60 s HIE, 60 s recovery; repeat 7x | 24:00 | 35 min MICT | 40:00 |
| 9 | 30 s HIE, 60 s recovery; repeat 6x followed by 15 s HIE, 45 s recovery; repeat 5x | 24:00 | 40 min MICT | 45:00 |
| 10 | 60 s HIE, 60 s recovery; repeat 5x followed by 15 s HIE, 45 s recovery; repeat 5x | 25:00 | 40 min MICT | 45:00 |

All HIT sessions commenced and concluded with a 5 min period of low-intensity cycling. MICT sessions commenced and concluded with a 5 min period of low-intensity cycling during week 1 and 2, which was reduced to 2.5 min from week 3 onwards. This time is included in the session time. *HIE*, high intensity exercise.
